# Inspection of thermal jump conditions on nanofluids with nanoparticles and multiple slip effects

**DOI:** 10.1038/s41598-022-07655-w

**Published:** 2022-04-04

**Authors:** Syed Muhammad Raza Shah Naqvi, Umar Farooq, Hassan Waqas, Taseer Muhammad, Ahmad Alshehri

**Affiliations:** 1 Department of Mathematics, Government College University Faisalabad, Faisalabad, 38000 Pakistan; 2 School of Energy and Power Engineering, Jiangsu University, Zhenjiang, 212013 China; 3 Department of Mathematics, College of Sciences, King Khalid University, Abha, 61413 Saudi Arabia; 4 Department of Mathematics, Faculty of Science, King Abdulaziz University, Jeddah, 21589 Saudi Arabia

**Keywords:** Energy science and technology, Engineering, Mathematics and computing, Nanoscience and technology

## Abstract

The significance of slip boundary conditions with thermal radiation implications on a steady flow of nano suspension over a rotating disk with a constant magnetic field is discussed in this research investigation. Here Iron oxide $\left( {Fe_{3} O_{4} } \right)$, Zirconium dioxide $\left( {ZrO_{2} } \right)$, and Titanium $\left( {Ti} \right)$ are recruited as nanoparticles and water $\left( {H_{2} O} \right)$ as a host fluid. The appropriate similarities transformations are used to transfer main PDEs into a system of nonlinear ODEs. The set of ODEs is then solved via shooting approach (bvp4c solver) a built-in function in MATLAB. The depictical outcomes of the physical flow parameters like thermal radiation and velocity slip parameters are revealed and clarified with the assist of figures. The slip parameter significantly reduces the velocity profiles, according to this investigation. The pressure is declined for the higher estimates of the magnetic parameter. The thermal profile was uplifted for the rising values of the thermal radiation parameter. Meteorology, meteorological, atmospheric research, biochemical engineering, power engineering, transportation production, solar energy transformations, sensing micro fabrication, tumblers in polymer manufacturing, and other fields will benefit from this suggested model. The suggested study has been developed in response to these kinds of practical consequences. This work is unique in that it investigates the consequences of a magnetic field, slip boundary conditions, and thermal radiation on nanoparticles flow across a disk. The recent study is innovative, and it could be used by other researchers to learn more about the heat exchange behavior and reliability of working fluids.

## Introduction

Nanofluids are a combination of nanoparticles and host fluid. Colloidal concentrations of nanoparticles in a base liquid form these. Low thermal conductivity is present in such base fluids. Because of their creation, nanoparticles are being used to enrich the efficiency of heat transportation in base fluids. They also help to increase heat capacity. Base fluids have a very low thermophysical phenomenon. Nanoparticles are used to increase the intensity of heat transmission in a base fluid due to their production; they also contribute to the increase in thermal physical phenomena. They have distinct chemical and physical characteristics. Following the pioneering work done in this area, tremendous development has occurred through Choi^1^. Eshgarf et al.^2^ explored the maximum energy consumption, and a study of the characteristics, preparation, modeling, and stabilization of hybrid nanofluids was presented. Sathyamurthy et al.^3^ analyzed nanofluids used in a study to cool a photovoltaic panel. Using the modified elliptical equation, Wakif et al.^4^ examines the impact of thermal radiation on the stability of hybrid nanosuspension. Nanofluids in concentrator collectors: Significant innovations and possibilities were presented by Buongiorno et al.^5^. The heat transfer and entropy production with novel $Co_{3} O_{4}$ hybrid nanofluids were presented by Said et al.^6^. Giwa et al.^7^ scrutinized the outcomes of base suspension, heat, and concentration on curve fitting. Hashemi et al.^8^ demonstrated that helix double-pipe heat is transferred, laminar thermal gradient, and flow properties of two distinct hybrid nanofluids using a novel curved conical tabulator. The impact of heat on hybrid nanofluids was studied by Wole-Osho et al.^9^. Using a new vortex generator, Ajarostaghi et al.^10^ examined the computational simulation of turbulent flow and heat transport of hybrid nanoparticles in a pipe. The renewable energy period is currently one of the most difficult and critical problems that civilization is challenged with. Solar electricity is a cost-effective solution to this problem. Solar power is also a natural way to generate electricity and energy. Solar energy is transferred in the form of thermal radiation, which is crucial for a variety of technical purposes, such as advanced power stations, gas-cooled nuclear reactors, and gas turbines. The importance of heat transmission by thermal radiation in the design of relevant devices cannot be overstated. Radiative outcomes are used to execute the heat transport procedure in the compound procedure industry. In the previous few years, a great amount of research has been done on guiding, convectional heat exchange, modeling, and associated programs. The performance of nanofluids can be extended by embedding more than one nanoparticle with the base fluid, resulting in a hybrid nanofluid. Hybrid nanofluids are now being studied numerically and experimentally. Hussain et al.^11^ studied the thermal radiation phase; the heat transmission of a hybrid nanofluid was investigated. Wakif et al.^12^ investigated the generalized Buongiorno nano liquid model, and we deliberated the impacts of thermal radiation and surface quality on hybrid nanosuspension. Muhammad et al.^13^ explored the interaction of the Jeffery nanofluid movement with the crossflow and the significance of variable thermal conductivity. Muhammad et al.^14^ looked at modeling elements of melting in nanofluids produced with thermal radiation over the sheet. Huang et al.^15^ investigated the thermal energy shielding properties of transparent Gd_2_Zr_2_O_7_/GdMnO_3_ thermally conductive polymers. Mesgarpour et al.^16^ investigated the use of solar panels for cooling: computational implementation of the new concept in porous materials for heat radiation. Ijaz et al.^17^ investigated the effects of thermal conductivity on ferromagnetic fluid flow. The dynamically adjustable surface transmittance is employed to construct dynamic thermal radiation mechanisms of action, according to Zeng et al.^18^. Waqas et al.^19^ analyzed the flow of crossing nanoparticles with thermal radiation, kinetic energy, and the melting mechanism. Natural convection flow in a restricted domain: electrohydrodynamics and radiative heat effects, Roy et al.^20^. Food processing, paper manufacturing, and wire and fiber treatment are all instances of non-Newtonian fluid flows generated by a stretched sheet that has been widely researched. In such processes, the cooling rate in the heat transfer process has a major impact on the quality of the completed product. One of the most important features for regulating the cooling rate and creating a high-quality product is the MHD parameter. The spectral theory for Casson fluid flow in a channel at MHD was discovered by Sheikh et al.^21^. The hybrid nanofluid flows were represented by Krishna et al.^22^ as a radiative MHD flow across an infinite exponential able to stand up the porous surface. Haq et al.^23^ investigated the chemical reaction and increasingly heated heat exchange mass and heat transfer, as well as MHD flow across a vertical plate. Using a Galerkin method, Hamid et al.^24^ investigated MHD nanofluid flow over channels. The form influence of MHD on Ferro-Brinkman-type nanofluids was calculated by Saqib et al.^25^. The reflect of heat production and absorption on the MHD flow of hybrid nanofluids above a bidirectional exponential sheet was studied by Zainal et al.^26^. An investigation of entropy production in MHD water flow over an advancing plate was explored by Abdelhameed^27^. Convective MHD flow modeling using hybrid powders was investigated by Shafee et al.^28^. Dawar et al.^29^ studied the MHD flow of Williamson nanosuspension across a nonlinear extended plate with chemical potential. Kumar et al.^30^ examined MHD flow and how heat is transported across porous discs in a laminar fashion. More work on nanofluids and nanoparticles is carried out^31–41^.

The goal of this study is to explore the importance of slip boundary conditions on nanofluid flow and thermal radiation across a rotating disk. This model is used in this work to explore the aspect of different Thermophysical properties of nanoparticles [Iron oxide $\left( {Fe_{3} O_{4} } \right)$, Zirconium dioxide $\left( {ZrO_{2} } \right)$, and Titanium $\left( {Ti} \right)$) and base fluid (Water $\left( {H_{2} O} \right)$]. The shooting approach is used with bvp4c solver built-in MATLAB to solve numerical solutions of dimensionless ODEs. The unique findings of the current analysis are helpful and valuable in academic study and various electrical, mechanical, and computer manufacturing processes like centrifugal filtration, gas turbine rotors, etc.

## Physically and mathematically flow modeling

### Flow description

**Figure 1:**
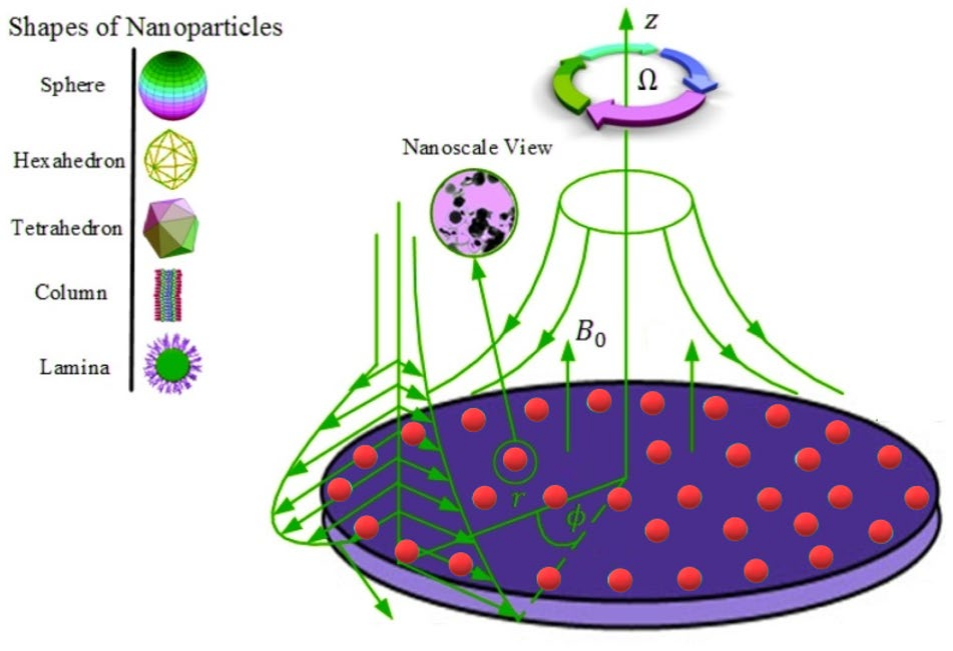
Flow geometry of considered problem.

A two-dimensional steady flow of nanofluid with nanoparticles (Iron oxide $\left( {Fe_{3} O_{4} } \right)$, Zirconium dioxide $\left( {ZrO_{2} } \right)$, and Titanium $\left( {Ti} \right)$) via a disk in the existence of slip boundary conditions and thermal radiation is considered. The cylindrical coordinates system is $\left( {r,\varphi ,z} \right)$. The $\left( {u,w} \right)$ are components of velocity in the directions of $\left( {r,\varphi ,z} \right)$ as shown in Fig. 1. In the axial direction of the disk, a magnetic strength $\left( {B_{0} } \right)$ of constant intensity is supplied. Additionally, we may ignore the induced magnetic field by assuming a low magnetic Reynolds number. In the existence of a phase nanofluid model, heat transport is also integrated at the disk's surface.

### Dimensional nonlinear equations

The main governing PDEs are (Iqbal et al.^42^):

1 $$u_{r} + \frac{u}{r} + w_{z} = 0,$$

2 $$ \rho_{nf} \left( {uu_{r} + \frac{{v^{2} }}{r} + wu_{z} } \right) = - p_{r} + \mu_{nf} \left( {u_{rr} + \frac{1}{r}u_{r} - \frac{u}{{r^{2} }} + u_{zz} } \right) - \sigma_{nf} B_{0}^{2} u, $$

3 $$\rho_{nf} \left( {uv_{r} + \frac{uv}{r} + wv_{z} } \right) = \mu_{nf} \left( {v_{rr} + \frac{1}{r}v_{r} - \frac{v}{{r^{2} }} + v_{zz} } \right) - \sigma_{nf} B_{0}^{2} v,$$

4 $$ \rho_{nf} \left( {uw_{r} + ww_{z} } \right) = - p_{r} + \mu_{nf} \left( {w_{rr} + \frac{1}{r}w_{r} + w_{zz} } \right), $$

5 $$\left( {\rho C_{p} } \right)_{{nf}} \left( {uT_{r} + wT_{z} } \right) = k_{{nf}} \left( {T_{{rr}} + \frac{1}{r}T_{r} + T_{{zz}} } \right) - q_{{r_{z} }} ,$$

With

$$ q_{r} = - \frac{{4\sigma^{*} }}{{3k^{*} }}T_{z}^{4} = - \frac{{16\sigma^{*} }}{{3k^{*} }}T^{3} T_{z} . $$

### Boundary conditions

With boundary constraints

6 $$\left. \begin{gathered} u = L_{1} u_{z} ,v = L_{1} v_{z} + r\Omega ,w = 0,T = T_{w} + L_{2} T_{z} \,at\,z = 0, \hfill \\ u \to 0,\,\,\,\,\,\,\,\,v \to 0,\,\,\,\,\,\,T \to T_{\infty } ,\,\,\,\,p \to p_{\infty } \,\,\,\,as\,\,\,\,\,\,\,z \to \infty \hfill \\ \end{gathered} \right\}.$$

### Transformation variables

The following transformation variables are

7 $$ \left. \begin{gathered} \zeta = z\sqrt {\frac{{U_{0} }}{{rv_{f} }}} u = r\Omega f^{\prime}\left( \zeta \right),v = r\Omega g\left( \zeta \right),w = - 2\sqrt {\Omega v_{f} } g\left( \zeta \right), \hfill \\ p = p_{\infty } - \Omega \mu_{f} P\left( \zeta \right),T = T_{\infty } + \left( {T_{w} - T_{\infty } } \right)\theta \left( \zeta \right) \hfill \\ \end{gathered} \right\}. $$

Here, $\left( {u,v\& w} \right)$ velocity components $\left( {\rho_{nf} } \right)$ are density, $\left( {\mu_{nf} } \right)$ is dynamic viscosity, and $\left( {\sigma_{nf} } \right)$ its electrical conductivity of nanofluid, $\left( {L_{1} } \right)$ wall slip coefficient, $\left( {L_{2} } \right)$ temperature jump coefficient, $\left( P \right)$ is pressure, and $\left( {U_{0} = \Omega r} \right)$ free stream velocity, respectively.

### Dimensionless equations

The dimensionless results of the main governing equations are

8 $$2\frac{{v_{nf} }}{{v_{f} }}f^{\prime\prime\prime} - f^{{\prime}{2}} + g^{2} + 4ff^{\prime\prime} - \frac{{\rho_{f} }}{{\rho_{nf} }}M^{2} f^{\prime} = 0,$$

9 $$2\frac{{v_{nf} }}{{v_{f} }}g^{\prime\prime} + 2fg^{\prime} - 2f^{\prime}g - \frac{{\rho_{f} }}{{\rho_{nf} }}M^{2} g = 0,$$

10 $$ \frac{{v_{nf} }}{{v_{f} }}f^{\prime\prime} + ff^{\prime\prime} - \frac{{\rho_{f} }}{{\rho_{nf} }}\frac{dP}{{d\zeta }} = 0, $$

11 $$\frac{{\left( {\rho C_{p} } \right)_{f} }}{{\left( {\rho C_{p} } \right)_{nf} }}\left( {\frac{{k_{nf} }}{{k_{f} }} + Rd} \right)\theta^{\prime\prime} + \Pr f\theta^{\prime} = 0,$$

With

12 $$\left. \begin{gathered} f\left( 0 \right) = 0,f^{\prime}\left( 0 \right) = \alpha f^{\prime\prime}\left( 0 \right),g\left( 0 \right) = 1 + \alpha g^{\prime}\left( 0 \right),\theta \left( 0 \right) = 1 + \beta \theta^{\prime}\left( 0 \right), \hfill \\ f^{\prime} \to 0,\,\,\,\,\,\,\,\,\,\,\,g \to 0,\,\,\,\,\,\,\,\,\,\,\,P \to 0,\,\,\,\,\,\,\,\,\,\,\theta \to 0\,\,\,\,\,\,\,\,\,\,\,\,\,When\,\,\,\,\zeta \to \infty \hfill \\ \end{gathered} \right\}.$$

### Reduced parameters

13 $$\left. {\left( {\Pr = \frac{{\mu_{f} \left( {C_{p} } \right)_{f} }}{{k_{f} }}} \right),\left( {M^{2} = \frac{{\sigma_{nf} B_{0}^{2} }}{{\Omega \rho_{f} }}} \right),\left( {\alpha = L_{1} \sqrt {\frac{\Omega }{{v_{f} }}} } \right),\left( {\beta = L_{2} \sqrt {\frac{\Omega }{{v_{f} }}} } \right),\left( {Rd = \frac{{4\sigma T_{\infty }^{3} }}{{k^{*} k_{f} }}} \right)} \right\}.$$

Here $\left( M \right)$ is the magnetic parameter, $\left( {Rd} \right)$ is the thermal radiation parameter, $\left( \beta \right)$ is the thermal slip parameter,$\left( \alpha \right)$ is the velocity slip parameter, and $\left( {\Pr } \right)$ is the Prandtl number.

The engineering parameters are:

14 $$\left. \begin{gathered} C_{f} \left( { = \frac{{\sqrt {\tau_{r}^{2} + \tau_{\theta }^{2} } }}{{\rho_{nf} \left( {r\Omega } \right)^{2} }}} \right), \hfill \\ Nu_{r} \left( { = \frac{{k_{nf} }}{{k_{f} }}\frac{{rq_{w} }}{{\left( {T_{w} - T_{\infty } } \right)}}} \right) \hfill \\ \end{gathered} \right\}.$$

The dimensionless results of engineering parameters

15 $$\left. {\left. {\tau_{w} = \mu_{nf} \left( {u_{z} + w_{r} } \right)} \right|_{z = 0} ,\left. {\tau_{\theta } = \mu_{nf} \left( {v_{z} + w_{r} } \right)} \right|_{z = 0} ,\left. {q_{w} = - k_{nf} \left( {T_{z} } \right)} \right|_{z = 0} } \right\}.$$

16 $${\text{Re}}_{r}^{\frac{1}{2}} C_{f} = \frac{{\mu_{nf} }}{{\mu_{f} }}\left( {f^{\prime\prime}\left( 0 \right)^{2} + g^{\prime}\left( 0 \right)^{2} } \right)^{\frac{1}{2}} ,$$

17 $$ {\text{Re}}_{r}^{{ - \frac{1}{2}}} Nu_{r} = - \frac{{k_{nf} }}{{k_{f} }}Rd\theta^{\prime}\left( 0 \right), $$

Here ${\text{Re}}_{r} \left( { = \frac{{2\Omega r^{2} }}{{v_{f} }}} \right)$ is the local Reynolds number.

### Numerical scheme

The flow model's system of ODEs (08–11) with boundary conditions (12) is investigated numerically using the efficacy and strength of numerical computing in terms of the Lobatto IIIA (bvp4c) technique and the computer tool MATLAB. The generated graphical results demonstrate the difference of momentum, pressure, and temperature profiles versus different physical factors. Ordinary differential equations system (08–11) transformed to first-order ordinary differential equations for a solution using Lobatto IIIA.

Let

18 $$\left. \begin{gathered} f = q_{1} ,f^{\prime} = q_{2} ,f^{\prime\prime} = q_{3} ,f^{\prime\prime\prime} = q^{\prime}_{3} ,g = q_{4} ,g^{\prime} = q_{5} ,g^{\prime\prime} = q^{\prime}_{5} , \hfill \\ P = q_{6} ,P^{\prime} = q^{\prime}_{6} ,\theta = q_{7} ,\theta^{\prime} = q_{8} ,\theta^{\prime\prime} = q^{\prime}_{8} \hfill \\ \end{gathered} \right\},$$

19 $$q^{\prime}_{3} = \left( {q_{2}^{2} - q_{4}^{2} - 4q_{1} q_{3} + \frac{{\rho_{f} }}{{\rho_{nf} }}M^{2} q_{2} } \right)\frac{{v_{f} }}{{2v_{nf} }},$$

20 $$q^{\prime}_{5} = \left( { - 2q_{1} q_{5} + 2q_{2} q_{4} + \frac{{\rho_{f} }}{{\rho_{nf} }}M^{2} q_{2} } \right)\frac{{v_{f} }}{{2v_{nf} }},$$

21 $$q^{\prime}_{6} = \left( { - \frac{{v_{nf} }}{{v_{f} }}q_{3} - q_{1} q_{3} } \right)\frac{{\rho_{nf} }}{{\rho_{f} }},$$

22 $$q^{\prime}_{8} = \frac{{ - \Pr q_{1} q_{8} \frac{{\left( {\rho C_{p} } \right)_{nf} }}{{\left( {\rho C_{p} } \right)_{f} }}}}{{\left( {\frac{{k_{nf} }}{{k_{f} }} + Rd} \right)}},$$

With

23 $$\left. \begin{gathered} q_{1} \left( 0 \right) = 0,q_{2} \left( 0 \right) = \alpha q_{3} \left( 0 \right),q_{4} \left( 0 \right) = 1 + \alpha q_{5} \left( 0 \right),q_{7} \left( 0 \right) = 1 + \beta q_{8} \left( 0 \right), \hfill \\ q_{2} \to 0,\,\,\,\,\,\,\,\,\,\,q_{4} \to 0,\,\,\,\,\,\,\,\,\,\,\,q_{6} \to 0,\,\,\,\,\,\,\,\,\,\,\,q_{7} \to 0\,\,\,\,\,\,\,\,\,\,\,\,\,When\,\,\,\,\zeta \to \infty \hfill \\ \end{gathered} \right\}.$$

## Results and discussion

**Figure 2:**
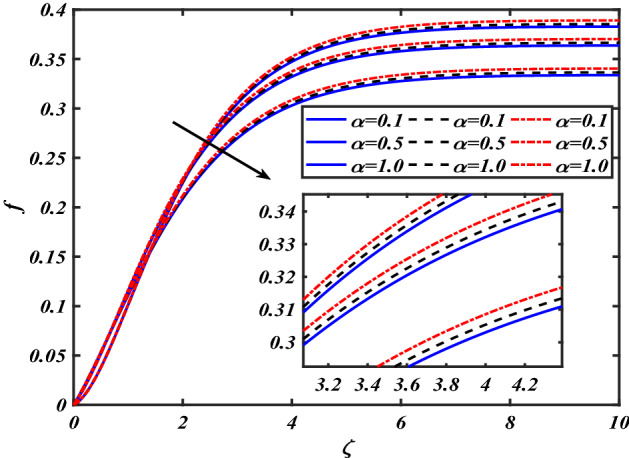
Pictogram of $\alpha$ via $f$.

**Figure 3:**
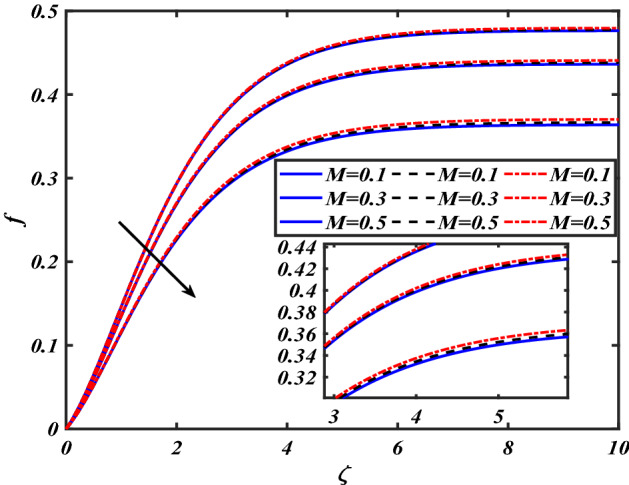
Pictogram of $M$ via $f$.

**Figure 4:**
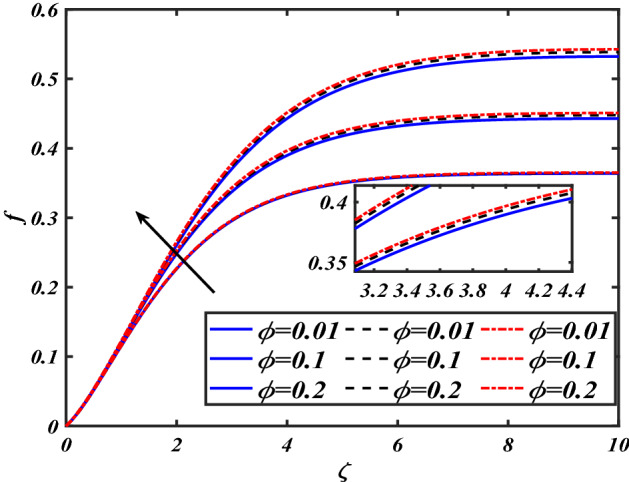
Pictogram of $\phi$ via $f$.

**Figure 5:**
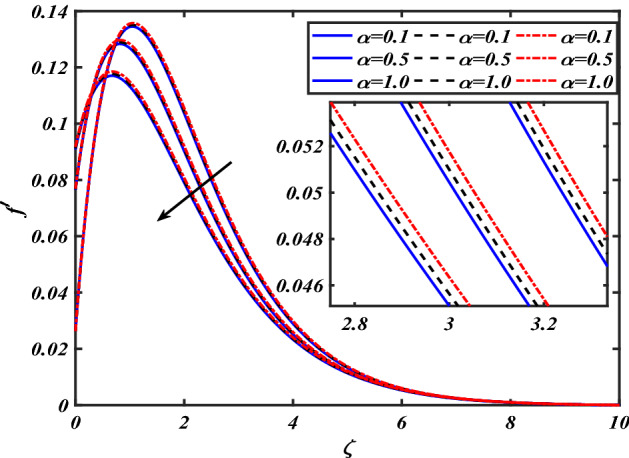
Pictogram of $\alpha$ via $f^{\prime}$.

**Figure 6:**
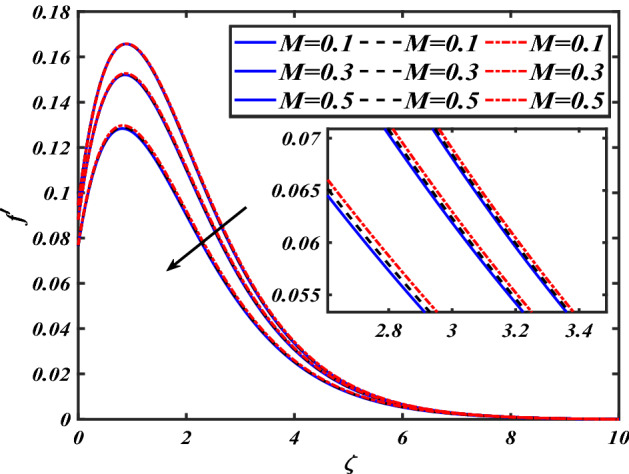
Pictogram of $M$ via $f^{\prime}$.

**Figure 7:**
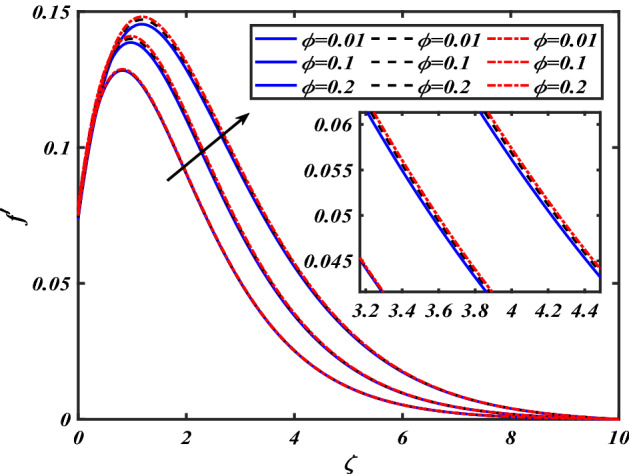
Pictogram of $\phi$ via $f^{\prime}$.

**Figure 8:**
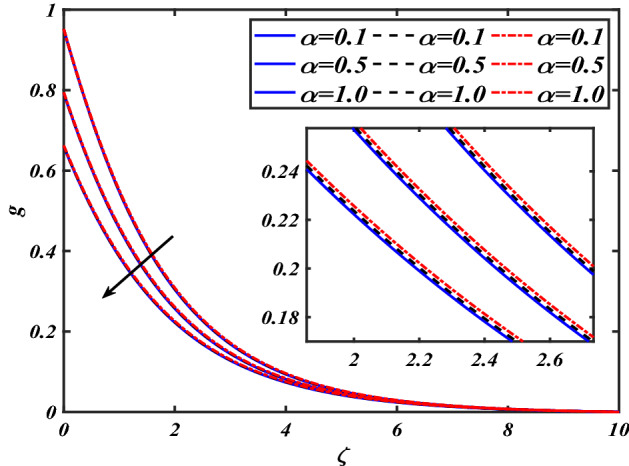
Pictogram of $\alpha$ via $g$.

**Figure 9:**
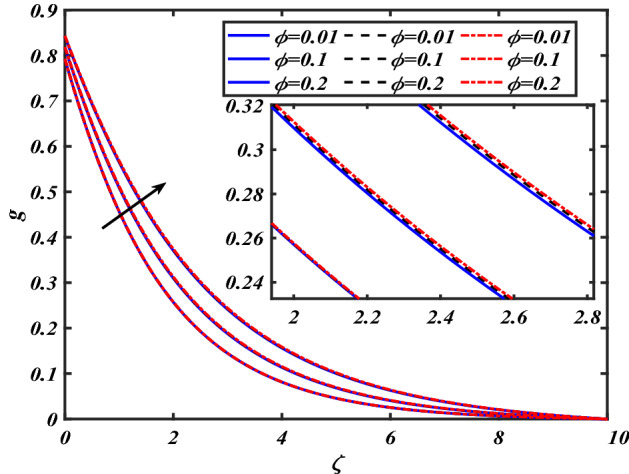
Pictogram of $\phi$ via $g$.

**Figure 10:**
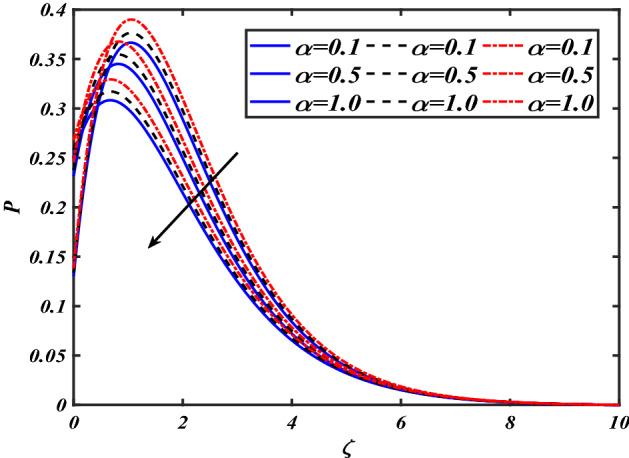
Pictogram of $\alpha$ via $P$.

**Figure 11:**
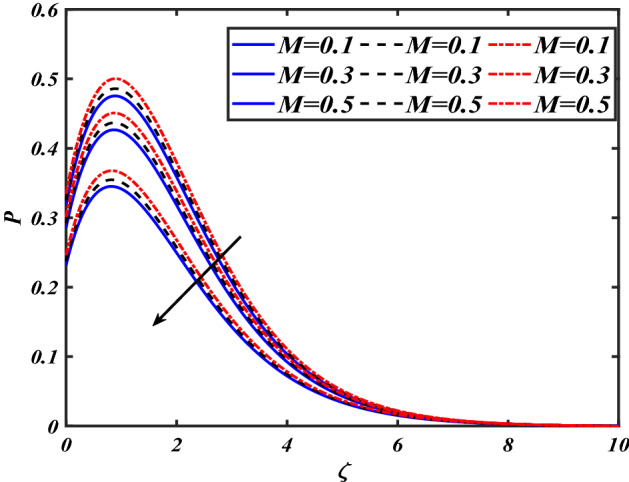
Pictogram of $M$ via $P$.

**Figure 12:**
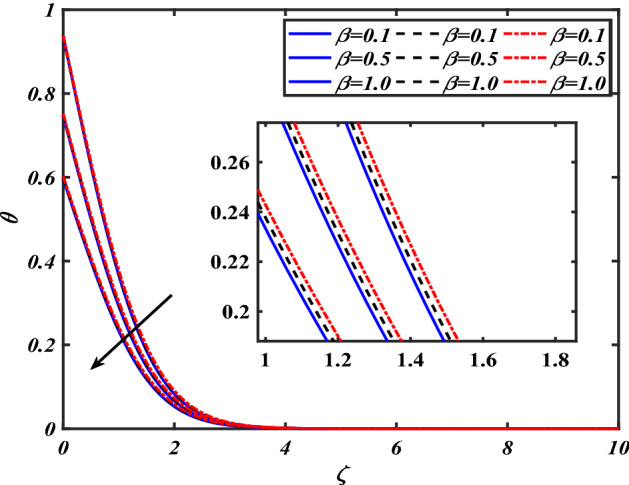
Pictogram of $\beta$ via $\theta$.

**Figure 13:**
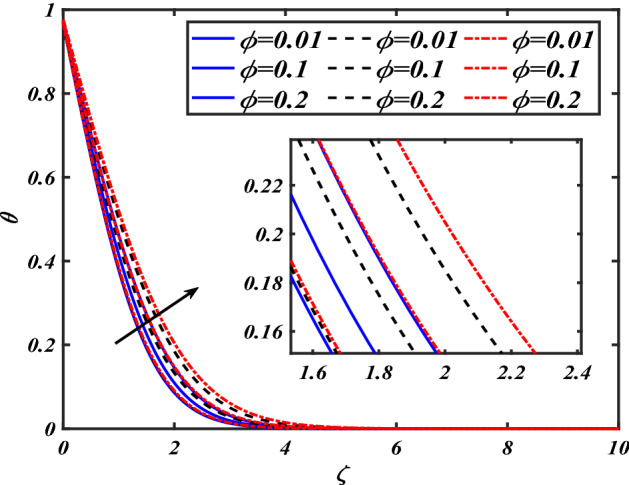
Pictogram of $\phi$ via $\theta$.

**Figure 14:**
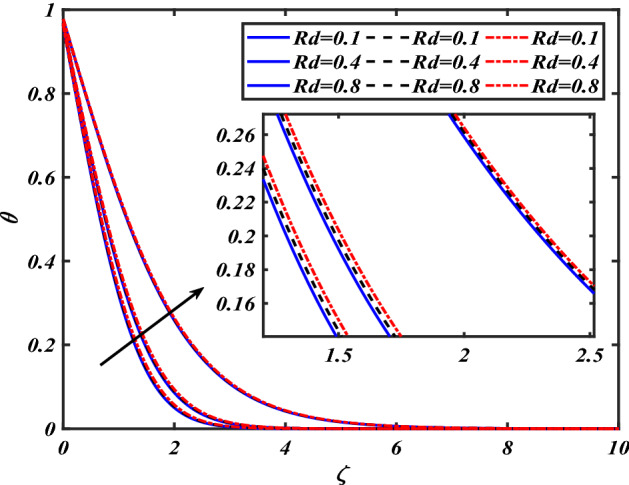
Pictogram of $Rd$ via $\theta$.

**Figure 15:**
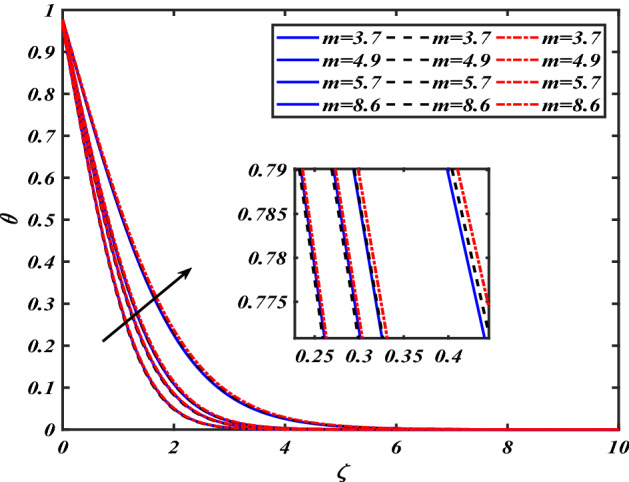
Pictogram of $m$ via $\theta$.

This section depicts the graphical results of flow parameters versus velocities and temperature and pressure. The dynamic of numerous components associated with the established flow pattern is greatly essential to investigate the insight physical features. The present section aims to assess the physical outcomes for important parameters on momentum, pressure, and temperature profiles. Tables are commonly used to depict the Thermophysical properties of nanoparticles. We have chosen fixed variations of non-dimensional variables $0.1 < \alpha < 1.0$,$0.1 < M < 0.5$,$0.01 < \phi < 0.2$,$0.1 < \beta < 1.0$, and $0.1 < Rd < 0.8$ for computational purposes. Here we discussed the aspects of different nanoparticles with a base fluid-like the blue solid line is for iron oxide $\left( {Fe_{3} O_{4} } \right)$, black line for Titanium $\left( {Ti} \right)$, and red line for the values of zirconium dioxide $\left( {ZrO_{2} } \right)$ with base fluid water $\left( {H_{2} O} \right)$. Figure 2 noted the influence $\alpha$ on the flow gradient $f$. The flow panel $f$ of the momentum boundary layer diminished as the velocity slip $\alpha$ was augmented. The aspect of $M$ the on velocity profile $f$ is demonstrated in Fig. 3. It is shown that both nanomaterials velocity $f$ are reduced for mounting approximations of magnetic parameters $M$. The physical nature of the Lorentz force was agreed upon by the graphical trends. However, it has been shown that adding copper to titanium causes a certain degree of drag force to be overcome. Figure 4 demonstrates the feature of $\phi$ the velocity profile $f$. The augment in the $\phi$ liquid enhances the axial velocity profile $f$. Figure 5 depicts the impact velocity slip parameter $\alpha$ $f^{\prime}$. The radial flow $f^{\prime}$ is reduced as the velocity slip parameter $\alpha$ is improved. The consequence magnetic strength $M$ on the radial velocity profile $f^{\prime}$ is established in Fig. 6. The hybrid momentum boundary layer $f^{\prime}$ falls for a mounting estimate of the $M$. As the magnetic parameter rises, so does the induced Lorentz force in the boundary layer, and therefore the velocity characteristics in the boundary layer. This suggests that with the increasing value of $M$, enhances the Lorentz force, and hence a rise in the Lorenz force resists the flow and inhibits fluid mobility. Furthermore, changing the magnetic parameter $M$ has no discernible influence on the thickness of the hydraulic boundary layer. Figure 7 reveals the aspect of the volume fraction of nanomaterials $\phi$ on.$f^{\prime}$. The augment in $\phi$, enhances the $f^{\prime}$. Figure 8 reveals the influence of $\alpha$ the velocity profile $g$. A decline in the velocity profile $g$ is noticed due to the hike in the velocity slip parameter $\alpha$. Figure 9 demonstrates the aspect of $\phi$ the flow profile $g$. The rise in the $\phi$ liquid enhances the velocity profile $g$. Figure 10 is sketched to highlight the aspect of velocity slip $\alpha$ on pressure $P$. It seems that the pressure $P$ decreases with increasing values $\alpha$. Figure 11 is plotted to highlight the features of magnetic parameter $M$ on pressure $P$. It appears that the pressure $P$ decays down with growing values of magnetic parameters $M$. Figure 12 depicts the effects of the thermal slip parameter $\beta$ on the temperature profile $\theta$. It is evident that the larger strength $\beta$ refuses the flow layer and advances the temperature profile $\theta$. The effect of the $\phi$ on $\theta$ is demonstrated in Fig. 13. Here, the presentation of temperature profiles $\theta$ and their thermal boundary layer is greater for the higher $\phi$. The variation in the temperature profile $\theta$ for thermal radiation parameters $Rd$ is symbolized in Fig. 14. The temperature $\theta$ is augmented with increasing thermal radiation parameters $Rd$. The review of heat gradient $\theta$ is stored in the fluid owing to the frictional forces due to which the thermal layer width is augmented. Thermal radiation has been one of three methods for exchanging energy between bodies of different temperatures. The emission of electromagnetic fields from the substance characterizes thermal radiation. We find that increasing the thermal radiation enhances the thickness of the thermal boundary layer, which improves heat transmission. An increase in the $Rd$ improves $\theta$. Increased thermal radiation parameter values deliver more heat to the working fluid, increasing temperature and thermal boundary layer thickness. Figure 15 shows the effects of shape factors ($m = 3.7$ bricks, $m = 4.9$ cylinders, $m = 5.7$ platelets, and $m = 8.6$ blades) $\theta$. The temperature profile $\theta$ displays the increasing behavior for the shape factor $\left( {m = 3.7,4.9,5.7,8.6} \right)$. Here we discussed the effects of different nanoparticles with a base fluid-like as the blue solid line is for iron oxide $\left( {Fe_{3} O_{4} } \right)$, black dashes line for Titanium $\left( {Ti} \right)$ and red line for the values of zirconium dioxide $\left( {ZrO_{2} } \right)$ with base fluid water $\left( {H_{2} O} \right)$. Thermophysical characteristics of nanofluid are shown in Table 1. Thermophysical characteristics of nanofluid were examined in Table 2. Furthermore, Table 3 shows the forms of diverse geometries of nanoparticles, which correlate to dissimilar estimations of shape factors. Table 4 interprets the numerical verification of the findings against the current literature.

**Table 1:** Thermophysical characteristics of nanofluid (Iqbal et al.^43^).

Properties	Nanofluid
Viscosity	$\mu_{nf} = \frac{{\mu_{f} }}{{\left( {1 - \phi } \right)^{2.5} }}$
Density	$\rho_{nf} = \rho_{f} \left[ {\left( {1 - \phi } \right) + \phi \left( {\frac{{\rho_{s} }}{{\rho_{f} }}} \right)} \right]$
Thermal Conductivity	$\frac{{k_{nf} }}{{k_{f} }} = \frac{{k_{s} + \left( {m - 1} \right)k_{f} - \left( {m - 1} \right)\phi \left( {k_{f} - k_{s} } \right)}}{{k_{s} + \left( {m - 1} \right)k_{f} + \phi \left( {k_{f} - k_{s} } \right)}}$
Heat Capacity	$\left( {\rho C_{p} } \right)_{nf} = \left( {\rho C_{p} } \right)_{f} \left[ {\left( {1 - \phi } \right) + \phi \frac{{\left( {\rho C_{p} } \right)_{s} }}{{\left( {C_{p} } \right)_{f} }}} \right]$

Here, $\left( m \right)$ is the shape factor, $\left( {Cp} \right)_{nf}$ the specific heat of nanosuspension, $\left( {k_{nf} } \right)$ is the thermal conductivity of nanosuspension, $\left( {\rho_{s} } \right)$ solid nanoparticles, $\left( {k_{f} } \right)$ is the thermal conductivity, $\left( {Cp} \right)_{f}$ base fluid, $\left( {\rho_{f} } \right)$ the density of the fluid, and $\left( {k_{s} } \right)$ solid nanoparticles.

**Table 2:** Experimental description of thermophysical characteristics of nanoparticles and base fluid (Mukhtar et al.^44^) and (Eid et al.^45^).

Constituents/properties	Density $\rho$	Specific heat $C_{p}$	Thermal conductivity $k$
Iron oxide $\left( {Fe_{3} O_{4} } \right)$	5810	670	6
Titanium $\left( {Ti} \right)$	4500	522	21.9
Zirconium dioxide $\left( {ZrO_{2} } \right)$	5680	502	1.7
Water $\left( {H_{2} O} \right)$	997.1	4179	0.6130

**Table 3:** Geometrical appearance of nanoscale particles as a function of shape factor (Ghadikolaei et al.^46^).

Geometry				
Shape	3.7	4.9	5.7	8.6
Shape factor	Bricks	Cylinders	Platelets	Blades

**Table 4:** The numerical validations for varying $\Pr$ on $- \theta^{\prime}\left( 0 \right)$ in limiting case

	Ramya et al.^47^	Li et al. ^48^	Present work
$\Pr$	$- \theta^{\prime}\left( 0 \right)$	$- \theta^{\prime}\left( 0 \right)$	$- \theta^{\prime}\left( 0 \right)$
6.2	3.7715	3.7717	3.7719
6.3	3.2514	3.2579	3.2581
6.4	2.8278	2.8280	2.8282

## Conclusion

In this investigation, we have to analyze the steady flow of nanofluid with thermal radiation across a rotating disk. Here the impacts of iron oxide $\left( {Fe_{3} O_{4} } \right)$, Zirconium dioxide $\left( {ZrO_{2} } \right)$, and Titanium $\left( {Ti} \right)$ are used as nanomaterials and water $\left( {H_{2} O} \right)$ as a host fluid. The slip phenomenon on velocities, pressure and thermal profile $\left( {r\& z} \right)$ were revealed graphically. The important results of this work are restated as follows:

- The flow gradients are reduced for extensive magnitudes of the velocity slip parameter and magnetic parameter.
- The elevated variations of value friction of nanostructures uplift the velocity profiles.
- The pressure gradient declines for higher values of slip parameter and magnetic parameter.
- The heat panel is decreased for increasing deviation of the thermal slip parameter.
- The more significant extent of the thermal radiation parameter upsurge the thermal profile.
- The temperature gradient depicts raising behavior for shape factors ($m = 3.7$ bricks, $m = 4.9$ cylinders, $m = 5.7$ platelets, and $m = 8.6$ blades).

## Abbreviations

$$r,\varphi ,z$$: Cylindrical-coordinate
$$u,v,w$$: Velocity components
$$T$$: Fluid temperature
$B_{0}$: Constant magnetic field
$$\tau_{r}$$: Radial stress
$L_{1}$: Wall slip coefficient
$$P$$: Pressure
$$T_{w}$$: Wall temperatures
$$T_{\infty }$$: Ambient temperatures
$L_{2}$: Temperature jump coefficient
$\Omega$: Uniform angular velocity
$$M$$: Magnetic parameter
$\left( k \right)_{f}$: Base fluid thermal conductivity
$\left( \rho \right)_{nf}$: Density of nanofluid
$\left( k \right)_{s}$: Thermal conductivity of nonomaterial
$\left( \rho \right)_{f}$: Density of suspension
$\left( \mu \right)_{f}$: Viscosity of suspension
$\left( k \right)_{nf}$: Thermal conductivity of nanofluid
$\left( \rho \right)_{s}$: Density of nanomaterials
$\left( {C_{p} } \right)_{nf}$: Specific heat of nanosuspension
$\left( \mu \right)_{nf}$: Viscosity of nanosuspension
$\left( {C_{p} } \right)_{f}$: Specific heat of the host fluid
$v_{f}$: Kinematic viscosity of the host fluid
$\zeta$: Dimensionless space variable
$$\tau_{\theta }$$: Tangential stress
$\beta$: Thermal slip parameter
$g$: Dimensionless centrifugal velocity
$\phi$: Solid particle volumetric fraction
$f$: Dimensionless axial velocity
$$\tau_{w}$$: Heat flux
$\left( {C_{p} } \right)_{s}$: Specific heat of nanoparticles
$$\Pr$$: Prandtl number
$$\theta$$: Dimensionless temperature
$\alpha$: Velocity slip parameter
${\text{Re}}_{r}$: Local Reynolds number
$Nu_{r}$: Local Nusselt number
